# Impact of urban land use tax on carbon emission efficiency of urban construction land——Analysis based on panel data of 30 provinces in China

**DOI:** 10.1371/journal.pone.0299730

**Published:** 2024-05-24

**Authors:** Sibo Ren

**Affiliations:** Institution of Chinese Agricultural Civilization, Nanjing Agricultural University, Nanjing, China; Qufu Normal University, CHINA

## Abstract

Reducing urban carbon emissions is an important path for ecological civilization construction, which can be achieved through the adjustment of urban land use tax. Using provincial Panel data from 2011 to 2021, based on the analysis of urban carbon emission efficiency values using a non radial SBM model, the Tobit random effects panel model is used to explore the institutional impact of urban land use tax. The study found that urban land use tax has a significant positive promoting effect on carbon emission efficiency and shows certain regional differences. The eastern region is higher in overall efficiency and technical efficiency than the central and western regions, but the central region has the highest overall scale efficiency. At the same time, factors such as population urbanization, industrial structure, and energy-saving technology level will also have a certain impact on this effect. Based on the institutional effect of improving carbon emission efficiency, the article proposes corresponding countermeasures and suggestions from aspects such as tax rate levels, tax system adjustments, tax incentives, and differentiated regional arrangements.

## 1. Introduction

At present, promoting green and low-carbon development has become a global consensus, and China has also established the goal of carbon peaking and carbon neutrality. The carbon emissions problem that triggers global warming and leads to ecosystem destruction has become the main ecological environmental factor that restricts people’s pursuit of a better life. Reducing carbon emission intensity is an inevitable choice for ecological civilization construction [1]. Urban areas are the main areas that generate carbon emissions. The expansion of urban construction land has led to the occupation of green land such as farmland, which in turn damages the ecological environment. Among all types of land, construction land accounts for about 80% of carbon emissions, and this proportion still shows a further upward trend [2,3]. Effective regulation of carbon emissions from urban construction land is necessary, and land intensification is the most feasible and universal method to achieve low-carbon utilization, which is of great significance. Urban land use tax is a tax levied on units and individuals who use land, based on their actual occupied land area, to promote the scientific, reasonable, and efficient use of land through paid use. Urban land use tax is a broad green tax [4], which is an important supplement to environmental protection tax and natural resource tax, and has an important regulatory effect on carbon emissions. The improvement of carbon emission efficiency in construction land is essentially the low-carbon regulation of land, emphasizing the control of the output end. Therefore, the solution to this problem can be considered from two aspects: on the one hand, the input end, which is the input of various energy and land resources, and on the other hand, the output end, which mainly constrains carbon emissions through tax means. The research focuses on the role and impact of urban land use tax on construction land, That is the latter.

Research on regulating the carbon emission efficiency of construction land can also be divided into three perspectives: energy consumption perspective, land use perspective, and tax regulation perspective. Firstly, from the perspective of energy consumption, energy utilization efficiency is mainly measured by the ratio of energy input per unit area of construction land to carbon emissions. The basic viewpoint is that carbon emission efficiency can compensate for the weakness of insufficient consideration of carbon emissions as a cost to expected output in indicators such as total carbon emissions [5], and land urbanization has a significant impact on urban energy utilization efficiency [6]. Corresponding political references are provided from adjusting energy structure, optimizing industrial structure, or converting old and new kinetic energy. Secondly, from the perspective of land use, there are two main ways to improve the carbon emission efficiency of construction land: firstly, to tap the potential of existing construction land, adjust the land use structure [7,8], and improve land intensive utilization [9,10]; secondly, to regulate the increment of construction land, suppress the growth rate of construction land [11–13], and regulate land use change [14]. The plan and planning management methods for achieving these two adjustments have issues such as strong rigidity, high costs, and long cycles. In contrast, land tenure tax, as a financial tool with regulatory effects on land use, has flexible and timely characteristics. It can reflect government intervention and play a market role, making it a relatively ideal regulatory tool. This is not only determined by the nature of taxation itself, but also verified by the practice of many developed countries. Finally, there is the perspective of tax regulation, which can be divided into specialized environmental taxation and comprehensive environmental taxation. Current research has made some explorations, believing that comprehensive environmental taxation inhibits the improvement of carbon emission efficiency of construction land, and pointing out that the functional structure deviation of the environmental tax system is the direct reason for this problem [15]. From the perspective of comprehensive environmental taxation and the relationship between carbon emission efficiency of construction land in recent years, the conclusions drawn from the perspective of tax regulation are arbitrary and lack analysis of regional differences and time changes. Urban land use tax belongs to a category of comprehensive environmental taxation and is one of the most important means of regulating the use of existing land. Therefore, starting from the urban land use tax, this study attempts to fill the theoretical gap by examining its regulatory effect on the carbon emission efficiency of construction land over a long period of time and in different regions.

At present, China is in a critical period of promoting ecological civilization construction and real estate tax reform, and there is an urgent need to incorporate the impact of land use on carbon emissions into institutional design. Due to the largest and most representative carbon emissions generated by urban construction land, and the fact that urban land use tax can directly reflect the use status of urban construction land, it is necessary to study the carbon emission effects of urban land use tax to clarify the relationship between the two. From the perspective of collection history and scope, urban land use tax is the main type of land tenure tax in China’s green tax system. Its collection department can adjust the urban land use structure through encouraging or inhibitory measures and lead it towards a stock model that leans towards green ecology. On the basis of selecting appropriate models and variables to measure the carbon emission efficiency of construction land, revealing the impact of urban land use tax has great practical significance. The research results can provide decision-making reference for formulating a tax system to regulate the carbon emission efficiency of urban construction land. To achieve this contribution, two objectives are set here: first, to analyze the reasons that affect the carbon emission efficiency of construction land, and second, to analyze the impact of urban land use tax on the carbon emission efficiency of construction land. To achieve these two goals, the discussion would be devided in two parts. Firstly, the total efficiency, technical efficiency, and scale efficiency of each province in detail are going to be calculated. Secondly, carbon emission efficiency of construction land should be set as the dependent variable, urban land use tax as the key variable, with several contorl variables for regression analysis to clarify the functional relationship between the two.

In this study, the reasons for selecting Chinese enterprises as the research object mainly include three points: firstly, China’s urbanization rate increased from 51.83% to 64.72% between 2011 and 2021 (calculated according to data from the National Bureau of Statistics of the People’s Republic of China), with a huge increase that is rare in other countries. As a result, a large amount of land resources have been converted from arable land to construction land, generating a large amount of carbon emissions; Secondly, there is a significant gap in the development level among the eastern, central, and western regions of China, with significant differences in the positive and negative output ratios generated per unit of land. Additionally, each province has different personalized characteristics such as energy-saving technology level, population urbanization rate, and industrial structure; Thirdly, the current tax basis for urban land use tax still lacks consideration for the degree of land use intensification, which results in poor control and regulation of land use behavior, including carbon emission efficiency. But apart from direct carbon tax regulation, urban land use tax is the most important type of indirect green tax among all.

## 2. Materials and methods

### 2.1 Data sources

The data used in this research is sourced from official public materials such as the "China Urban Construction Statistical Yearbook", "China Statistical Yearbook", and "China Energy Statistical Yearbook". Regional carbon dioxide is calculated based on various major energy consumption by using provincial energy balance tables, standard coal conversion coefficients, standard coal heating coefficients, and IPCC values. For some regions where there is a lack of data in certain years, local yearbooks or data published by local statistical bureaus will be selected for supplementation.

### 2.2 Efficiency measurement method

#### 2.2.1 Efficiency model selection

To calculate carbon emission efficiency, we need to consider unexpected output and input-output slack. Compared with Data envelopment analysis (DEA) or Random Frontier Analysis (SFA), which are usually used to evaluate general static input-output, SBM model can better meet this requirement. This paper uses the research of Tone [16] as a model for calculating carbon emission efficiency of urban construction land, which can be expressed as:

$$\text{min}\;\rho =\frac{1-\frac{1}{m}\sum_{i=1}^{m}s_{i}^{-}/x_{ik}}{1+\frac{1}{q}\sum_{r=1}^{q}s_{r}^{+}/y_{rk}}$$

$$s_{.}t_{.}\;X\lambda +s^{-}=x_{k}$$ (1)

$$\text{Y}\lambda -s^{+}=y_{k}$$

$$\lambda ,\;s^{+},s^{-}\geq 0$$

In Eq (1), *s*^+^ and *s*^−^ separately represents the insufficient output and excess input in urban production activities, *λ* represents the optimal weight vector of input and output factors, *ρ* represents the carbon emission efficiency value of construction land, with a value of [0,1].*x*_*ik*_ represents the i-th input of the k-th province (autonomous region, municipality directly under the central government), *y*_*rk*_ represents the r-th output of the k-th province (autonomous region, municipality directly under the central government), and m and q separately represent the number of inputs and outputs.

#### 2.2.2 Indicator selection and its statistical values

Among various indicators, land investment selects the area of urban construction land, which is based on the reason that urban land use tax is levied for the preservation of urban construction land; Capital input mainly refers to the input of material capital. For the production activities of enterprises, some fixed assets are directly related to production activities. Among them, equipment fixed assets investment reflects the technical level and production capacity of enterprises, so urban fixed assets investment is selected to represent this variable. The variable of labor input is represented by the number of employees in the secondary and tertiary industries, including those engaged in real economy and virtual economy industries. Output indicators include expected output and unexpected output. Expected output, i.e. economic output, is selected as the variable of added value in the secondary and tertiary industries; Unexpected output, i.e. energy emissions that cause damage to the environment, using the indirect energy consumption method proposed by Wang Liping et al. [17] as a reference, based on the energy balance tables of each province and city for each year, five energy sources including raw coal, washed coal, crude oil, fuel oil, and natural gas are selected as benchmarks, and the annual carbon dioxide emissions of each province are calculated based on the method in the 2011 National Greenhouse Gas Inventory Guidelines.

According to the descriptive statistical values of each variable (Table 1), it can be seen that there are significant differences among various indicators in each province (autonomous region/municipality), with smaller mean distances and closer values. This indicates that there are a few provinces where the input-output ratio is much higher than that of other provinces, and it also indicates that while producing high economic income, high carbon emissions may have been generated, and carbon emission efficiency is still low. Therefore, specific analysis needs to be conducted through calculating efficiency. The construction land area in the input indicator and the carbon dioxide emissions in the output indicator are the two largest standard deviations, indicating that there are significant internal differences between these two types of variables. It is necessary to analyze them to discover their internal influencing factors and the relationship between the two, and propose relevant countermeasures and suggestions.

**Table 1:** Numerical statistical description of the evaluation index of carbon emission efficiency of construction land in 30 provinces of China.

Indicator layer	Min	Max	Mean	Std.Dev
Area of Construction land (hectares)	10776	495873	1415383	98257.1
Urban fixed assets investment (100 million yuan)	384.61	52364.5	10558.95	9345.44
Labor force in the second and third industries (10000 people)	82.37	5024.10	932.44	738.35
Value added of secondary and tertiary industries (100 million yuan)	571.94	77160.54	15280.73	13780.7
Carbon dioxide emissions (10000 tons)	1762.06	94897.22	30358.49	20960.72

### 2.3 Construction of efficiency impact model

#### 2.3.1 Construction of tobit model for random effects

Based on the aforementioned method, calculate the carbon emission efficiency of construction land in various provinces of China from 2011 to 2021 and use it as the dependent variable. Due to the fact that the efficiency variable is a constrained dependent variable with a value range of [0,1], in addition, the unobservable individual effects in resource factor endowments, economic development levels, and other aspects of each province will have an impact on the explanatory variables. There may be a situation where the carbon emission rate is negative (in cases where carbon absorption is greater than carbon emissions), achieving so-called "carbon neutrality", which will interfere with the judgment of carbon emission efficiency in general situations. Thus, the panel Tobit model with random effects can effectively describe the efficiency impact relationship (Represented here as the panel model of merged data):

$$\;y_{it}^{*}=x_{it}^{\prime }\beta +u_{i}+\varepsilon_{it}$$ (2)

$$y_{it}=\left\{\begin{array}{c} y_{it}^{*},\;if\;y_{it}^{*}>0\\ 0,if\;y_{it}^{*}\leq 0 \end{array}\right.$$ (3)

In this formula, *y*_*it*_ represents the carbon emission efficiency of construction land in region i in year t, $x_{it}^{'}$ represents various main influencing factors in region i in year t, *u*_*i*_ represents individual characteristics, and *ε*_*it*_ represents errors.

#### 2.3.2 Variable settings

The carbon emission efficiency of construction land and its corresponding technical efficiency and scale efficiency are set as the dependent variables, and urban land use tax is the key explanatory variable. Based on existing research, urbanization rate [18], industrial structure [19,20], energy-saving technology level [21,22], and government land transfer volume are selected as the control variables. Table 2 provides a concise list of variable classification, variable names, variable explanations, and variable units involved in regression analysis.

**Table 2:** Measurement model variable settings.

Variable classification	Variable	Variable Interpretation	Variable Units
Explained Variable	Overall efficiency	Carbon emission efficiency of construction land	/
	Technical efficiency	Combinatorial optimization degree of various production factors	/
	Scale efficiency	Investment scale of each element	/
Explanatory variable	Urban land use tax	Annual paid land use tax by each province	10 thousand yuan
	Urbanization rate	Urban population/Total population	%
	Industrial structure	Output value of Tertiary sector of the economy/Total output value of secondary and tertiary industries	%
	Energy saving technology level	Energy consumption per unit output value of secondary and tertiary industries	Tons/10 thousand yuan

The basis for calculating urban land use tax is land area, which is a key indicator for deriving the carbon emission efficiency of construction land. Therefore, urban land use tax is linked to the carbon emission efficiency of construction land in a certain proportion. The urbanization rate is measured at the population level, which refers to the proportion of urban population to the total regional population, and to some extent reflects the size of the potential carbon emission generating population in cities. The industrial structure is the proportion of urban tertiary GDP to the total GDP of the secondary and tertiary industries, compared to the second industry, the third industry has a more significant effect on reducing carbon emissions [23]. The ratio of its output value to the total output value of the second and third industries reflects the distribution of industry types and also has a certain explanatory power for carbon emission efficiency. The level of energy-saving technology can reflect the level of energy-saving and emission reduction technology of enterprises. Previous studies have mostly selected the authorized amount of technology patents, but this standard is weak in specificity and difficult to represent the level of energy-saving technology. Therefore, the energy consumption per unit output value of the secondary and tertiary industries is used to represent the level of energy-saving technology.

According to Table 3, from the descriptive statistical analysis of each variable, it can be seen that the maximum values of total efficiency, technical efficiency, and scale efficiency are all 1, indicating that there are effective situations. Among them, scale efficiency is relatively high, and technical efficiency needs to be further improved to improve the overall efficiency. There are significant regional differences in urban land use tax, with extreme outliers that are much higher or lower than the average. This is not only related to factors such as urban scale, land resource endowment, and financial pressure between regions, but also constrained by local construction land indicators, enterprise willingness, and other factors. Similarly, differences in urbanization rate, industrial structure, and energy-saving technology level are also significantly different due to differences in economic development levels in the region.

**Table 3:** Variables of the econometric model describe the statistical values.

Indicator layer	Min	Max	Mean	Std.dev
Overall efficiency	0.42	1	0.78	0.17
Technical efficiency	0.59	1	0.86	0.14
Scale efficiency	0.51	1	0.95	0.08
Urban land use tax (yuan)	900	3937400	355854	488661
Urbanization rate (%)	27.46	89.6	52.9	13.83
Industrial structure (%)	33.32	80.64	47.28	8.45
Energy saving technology level	0.23	7.52	1.113	0.732

## 3. Results and discussion

### 3.1 Efficiency calculation results

**Fig 1:**
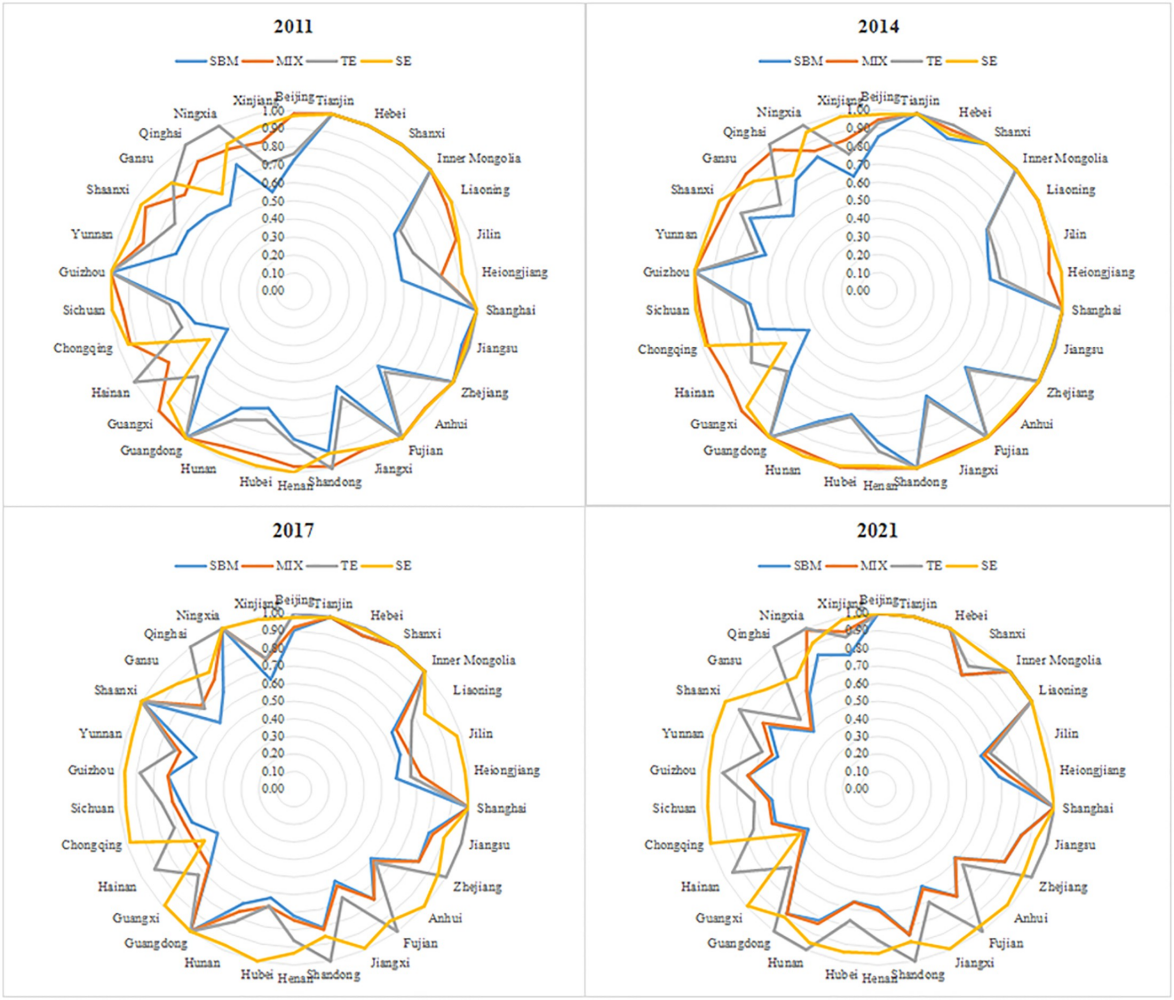
Carbon emission efficiency of construction land in China’s provinces from 2011 to 2021.

Using MAXDEA8.0 as a tool to calculate carbon emission efficiency, the total carbon emission efficiency(SBM), mixed efficiency(MIX), technical efficiency(TE), and scale efficiency(SE) of 30 provinces, autonomous regions, and municipalities directly under the central government in China were calculated, and the characteristics and changes of carbon emission efficiency in the eastern, central, and western regions were summarized. Represented by four years such as 2011, 2014, 2017, and 2021, certain changes in efficiency values can be obtained based on their characteristics.The reason for selecting these years as observation nodes is twofold: on the one hand, this period is around the 18th and 19th National Congress of the Communist Party of China, and there have been significant policy adjustments in energy conservation and emission reduction, as well as carbon emission trading quotas in 2014 and 2021, which have strong representativeness. On the other hand, considering that this period is closest to the current period, its results are more informative, and the data is easier to obtain, which has certain feasibility and reference value.The calculation result of efficiency value is shown in Fig 1, among them, SBM, MIX, TE, and SE are represented by different colored lines. The outer edge of the radar map is the name of the observed province, and the value range from 0 to 1 is from the center outward, the four graphs correspond to the selected time nodes 2011, 2014, 2017, and 2021.

In this series of efficiency, the total carbon emission efficiency (SBM efficiency) represents the actual efficiency value of using a certain amount of land, capital, and labor input to generate the maximum output of economic benefits and the minimum output of carbon dioxide emissions. Tianjin, Inner Mongolia, and Shanghai have always been in an effective state, while Hainan has always been the province with the lowest carbon emission efficiency, while Beijing and Liaoning provinces have maintained stable efficiency growth; Fujian in the eastern region, Henan in the central region, Guizhou and Gansu in the western region show a gradual decline trend. The carbon emission efficiency of the other four provinces, including Shandong, Hunan, Shaanxi, and Ningxia, shows a trend of fluctuating high and low.

According to the theory of efficiency and productivity, there are two reasons for the inefficiency of overall efficiency: firstly, technological inefficiency, and secondly, scale inefficiency. Technological inefficiency is the primary cause of overall efficiency inefficiency, with Anhui, Jiangxi, Hubei, and Northeast provinces as typical representatives. The typical representative provinces of ineffective scale are Hainan Province and Qinghai Province. These two provinces have relatively high technological efficiency, and Qinghai Province has even been in a state of strong and effective technological efficiency. However, the two provinces have the problem of being too small in terms of construction land, labor, and capital investment, and are far from moderate scale under conditions of strong and effective technology or relatively effective technology.

From the perspective of the eastern, central, and western regions, the total efficiency, technical efficiency, and scale efficiency of the provinces (autonomous regions, municipalities directly under the central government) included in each major region are summed up to obtain different types of efficiency in each region and year. From Table 4, it can be seen that in terms of overall efficiency, the eastern region>the central region>the western region, indicating that the eastern region is handling the relationship between production development and unexpected output properly. The three major regions showed a slow downward trend from 2014 to 2017, while the central and western regions continued to decline by 2021, which may be constrained by the difficulties of the original industrial transformation; In terms of technological efficiency, the eastern region>the western region>the central region, and the three regions have been increasing year by year, indicating continuous improvement and breakthroughs in technology and management. In addition, the phenomenon of the western region being slightly higher than the central region also indicates that the central region has poor ability to utilize resources; In terms of scale efficiency, the central region>the western region>the eastern region. Although the eastern region has the highest land intensive utilization, it has not shown better carbon emission scale efficiency. On the contrary, the central region utilizes its own advantages such as abundant land resources and combines local technologies to integrate various resources, improve scale efficiency, and ultimately increase overall efficiency.

**Table 4:** Summary of regional carbon emission efficiency.

Efficiency type	Region	2011	2014	2017	2021
Overall efficiency	Eastern region	0.88	0.90	0.84	0.87
	Central region	0.73	0.76	0.72	0.71
	Western Region	0.67	0.73	0.71	0.66
Technical efficiency	Eastern region	0.95	0.95	0.97	1.00
	Central region	0.79	0.78	0.78	0.81
	Western Region	0.82	0.81	0.82	0.81
Scale efficiency	Eastern region	0.94	0.95	0.91	0.92
	Central region	0.97	0.99	0.98	0.97
	Western Region	0.91	0.95	0.96	0.94

### 3.2 Tobit model measurement results and analysis

To better analyze the reasons for efficiency differences between provinces and between the East, West, and Central regions, total efficiency, technical efficiency, and scale efficiency are used as the explanatory variables. After determining the units of all explanatory variables, in order to avoid estimation errors caused by numerical differences between the data, logarithms were calculated for various types of efficiency, taxation, urbanization rate, industrial structure, and energy-saving technology level to smooth them out. From Table 5, it can be seen that taxation has a significant positive impact on the total efficiency value, but its coefficient is small. This indicates that the current provincial urban land use tax has a significant regulatory effect on the carbon emission efficiency of construction land, but its regulatory effect is still small and has not fully played the regulatory effect that urban land use tax should have. This is due to the low standard of urban land use tax amount. The direct relationship between urban land use tax and carbon emission efficiency of construction land is less closely related. From the results, it can be seen that urban land use tax has a significant positive impact on scale efficiency, indicating that urban land use tax is beneficial for enterprises to adjust their scale and generate higher scale efficiency through economic means. This is also the main path for urban land use tax to affect the carbon emission efficiency of construction land.

**Table 5:** Analysis results of provincial Tobit random panel model.

Variable	(1) lnte	(2) sigma_u	(3) sigma_e	(1) lnpte	(2) sigma_u	(3) sigma_e	(1) lse	(2) sigma_u	(3) sigma_e
lntax	0.018*** (0.006)			-0.000 (0.004)			0.006** (0.002)		
lnurb	0.607*** (0.064)			0.369*** (0.055)			0.107*** (0.035)		
lnis	-0.202** (0.102)			0.046 (0.082)			-0.238*** (0.052)		
lnel	0.095*** (0.021)			0.057*** (0.016)			0.005 (0.010)		
Cons	0.267*** (0.058)	0.022*** (0.008)	0.078*** (0.003)	0.405*** (0.040)	0.000 (0.005)	0.068*** (0.003)	0.692*** (0.025)	0.000 (0.004)	0.043*** (0.002)
N	330	330	330	330	330	330	330	330	330
Ids	30	30	30	30	30	30	30	30	30

Note:

***, **, *Significantly represent statistical values at the 1%, 5%, and 10% levels, respectively.

In addition, the urbanization rate and energy-saving technology level have significant positive effects on the carbon emission efficiency of construction land, while the industrial structure has a significant negative impact. Sigma_ U is significantly not zero, indicating that individual effects have a significant impact on overall efficiency, indicating significant individual differences between different provinces; In addition, urbanization rate and energy-saving technology level have a significant positive impact on technological efficiency, indicating that population urbanization may improve the management level of enterprises to a certain extent; In the regression of scale efficiency, urban land use tax and urbanization rate have a significant positive impact, while industrial structure has a significant negative impact. This shows that, on the one hand, with the increase of urban land use tax rate and urbanization level, the carbon emission efficiency of construction land under a certain scale tends to be higher. On the other hand, a large proportion of the output value of the Tertiary sector of the economy may mean that the scale efficiency is low. The industrial advantage of occupying more construction land itself has not been fully utilized, and there is still much room for improvement.

According to the regression results listed in Table 6, from the perspective of zoning, taxation has a significant and relatively small negative impact on technological efficiency. The carbon emission elasticity coefficient of lntax is -0.010, indicating that there may still be some issues with the regulatory ability of urban land use tax on land allocation between regions. It can be seen that the constant term has a significant negative impact on overall efficiency and technical efficiency, indicating that there must be other influencing factors between different regions. These factors are relatively good/abundant in the central and western regions, but have a negative impact on the carbon emission efficiency of construction land. The urbanization rate has a significant positive impact on both technological efficiency and overall efficiency.

**Table 6:** Regional regression results of eastern, central and western regions.

Variable	(1)	(2)	(3)	(1)	(2)	(3)	(1)	(2)	(3)
	lnte	sigma_u	sigma_e	lnpte	sigma_u	sigma_e	Lnse	sigma_u	sigma_e
lntax	-0.006			-0.010**			0.001		
	(0.006)			(0.004)			(0.002)		
lnurb	0.250***			0.174***			0.006		
	(0.029)			(0.030)			(0.014)		
lnis	0.101*			0.272***			-0.089***		
	(0.058)			(0.084)			(0.024)		
lnel	0.034			0.058**			-0.004		
	(0.024)			(0.026)			(0.012)		
Cons	-0.803***	0.024***	0.013***	-1.113***	0.014**	0.016***	0.986***	0.000	0.010***
	(0.192)	(0.008)	(0.002)	(0.297)	(0.007)	(0.003)	(0.099)	(0.004)	(0.001)
N	33	33	33	33	33	33	33	33	33
years	11	11	11	11	11	11	11	11	11

Note:

***, **, * respectively indicate significant statistical values at the 1%, 5%, and 10% levels.

From the impact of urbanization rate on technological efficiency, it can be seen that the improvement of urbanization rate is achieved by improving its technological efficiency, supplemented by the improvement of industrial structure and energy-saving technology to improve carbon emission efficiency. The industrial structure has a positive and negative impact on technical efficiency and scale efficiency respectively. The development of the Tertiary sector of the economy, especially the high-tech industry, will bring many clean and green technologies, which is conducive to minimizing carbon emissions under the conditions of maximum economic returns. The negative impact on the scale efficiency is the same as the reason at the provincial level. The constant term has a significant impact on all three, indicating that there are still some significant influencing factors that have not been considered and further exploration is needed. Additionally, individual differences have a significant impact on carbon emission efficiency.

### 3.3 Robustness testing

The regression analysis of model parameters examined the relationship between urban land use tax and carbon emission efficiency of construction land. In order to verify the stability and reliability of the conclusion, further robustness testing of the model is needed. This study set urbanization rate, industrial structure, and energy-saving technology level as control variables. In order to test the robustness of the model, comprehensive efficiency was selected as the independent variable. The carbon reduction effect of urban land use tax was re evaluated by increasing or decreasing the number of control variables. Table 7 reports the robustness results of urban land use tax on carbon emission efficiency of construction land, confirming that the former has a positive impact on the latter.

**Table 7:** Robustness test results.

variable	lnte			
	model (1)	model (2)	model (3)	model (4)
lntax	0.00713 (0.91)	0.0197** (2.63)	0.0192* (2.57)	0.0209** (2.78)
lnurb		-0.806*** (-0.716)	-0.839*** (-7.22)	-0.838*** (-7.23)
lnis			-0.0753 (-1.13)	-0.0705 (-1.06)
el				0.0179 (1.76)
Cons	-0.136*** (-4.66)	3.351*** (6.87)	3.821*** (5.96)	3.794*** (5.93)
N	330	330	330	330

## 4. Conclusion and policy recommendations

### 4.1 Research conclusion

On the basis of elaborating on the role of urban land use tax on carbon emission efficiency, this article constructs an SBM-DEA model and calculates the carbon emission efficiency of 30 provinces (municipalities, autonomous regions) in China from 2011 to 2021. It analyzes and explores some of China’s economic situation and policy background. Based on the calculated data and explanatory variables related to carbon emission efficiency, a panel random Tobit model is constructed. Regression analysis is conducted on the total efficiency, technical efficiency, and scale efficiency of each variable by province, east, central, and western regions, and the following conclusions are drawn:

1. At the provincial level, urban land use tax has a significant positive impact on overall carbon emission efficiency and scale efficiency, but has no significant impact on technological efficiency. The significant impact of this on overall efficiency and scale efficiency has not been fully utilized, and its powerful regulatory role has not yet been fully demonstrated. It is necessary to further strengthen tax reform efforts to regulate it. There are significant individual differences among provinces, and different provinces should introduce different policies based on local factor endowments and socio-economic environment to achieve the optimization of this regulation, emphasizing the introduction of incentive measures to promote technological efficiency improvement.
2. At the regional level, urban land use tax has a significant negative impact on carbon emission technology efficiency, while its impact on overall efficiency and scale efficiency is not significant. However, carbon emission efficiency shows significant regional differences, showing an overall trend of East>Middle>West. There is no significant interaction between urban land use tax and carbon emissions of construction land in the east and west. It is necessary to build a set of relevant systems that combine the two together. In addition, in terms of scale efficiency, the scale efficiency of the central region is better than that of the eastern and western regions. In terms of time progression, the overall efficiency and scale efficiency reached a peak before and after 2014. Therefore, considering that the western region is close to the optimal scale, while the central eastern region shows a decreasing trend, the overall technical efficiency shows a slow fluctuation and upward trend.
3. In addition to urban land use tax, there are many factors that cannot be ignored that have a significant impact on the carbon emission efficiency of construction land, such as the level of energy-saving technology, population urbanization, industrial structure, etc. The level of energy-saving technology has a significant impact on scale efficiency and overall efficiency at the provincial level, while it has a significant impact on technical efficiency at the eastern, central, and western regional levels. From a more macro perspective such as the eastern, central, and western regions, the level of energy-saving technology directly affects its ability to achieve minimum energy input under a given output. In addition, population urbanization has a significant positive impact on overall efficiency and technological efficiency, and the correlation is significant. But at the provincial level, it has a significant negative impact on overall efficiency. This indicates that population mobility has had a good factor allocation effect between regions. The industrial structure exhibits similar characteristics with more significant inter regional impacts, and there is a significant positive and negative correlation with technological efficiency and scale efficiency.

There are still certain limitations to the research, such as whether urban land use tax can design direct green environmental protection measures to regulate the carbon emission efficiency of construction land in addition to regulating land use intensity. In addition, what is the relationship between urban land use tax and farmland occupation tax, and how to coordinate the two to improve the overall carbon emission efficiency of land use. These are all areas that were not covered in this study and are also gaps that need to be filled in future research.

### 4.2 Policy recommendations

Through theoretical analysis and empirical testing, the above conclusions have important implications for improving the carbon emission efficiency of construction land:

1. At the provincial level, based on the economic development of each province, the level of urban land use tax rate should be correspondingly increased, and its resource allocation adjustment function should be strengthened. Improve the overall standard of urban land use tax, clarify the carbon emissions per unit of various types of construction land through a series of methods, and establish a set of tax incentives or penalties linked to the carbon emissions per unit of each type of construction land. Further clarify the loss of ecological Environmental Values caused by production activities on construction land, and include it into urban land use tax for compensatory collection.
2. At the regional level, efforts should be made to reconstruct the relationship between regional urban land use taxes and carbon emissions, and to implement differentiated regional arrangements. The method of linking urban land use tax with carbon emissions based on a certain percentage can better leverage the tax regulation effect on indirect carbon emissions from land use. From the perspective of technological efficiency and scale efficiency, on the one hand, promoting the introduction of advanced technology from the central and western regions to the east; on the other hand, provinces with lower scale efficiency, mainly represented by Hainan and Qinghai provinces, should focus on providing land transfer opportunities, financial support, and various social services for green and environmentally friendly industries according to their own situation.
3. In other aspects, improve supporting measures, including improving the level of energy-saving technology, population urbanization, and industrial structure. Local tax incentives should be used to attract and encourage professional talents and urban labor to move and optimize between provinces. At the same time, we will develop high-tech industries and clean energy industries through guiding policies, promote cleaner production and intensive development of enterprises, and formulate relevant policies to promote the transformation and upgrading of industrial structure, so as to improve the carbon emission efficiency of urban construction land.

## Supporting information

S1 Dataset (XLSX)

## Abbreviations

Cons: constant
el: energy saving technology level
ids: intrusion Detection Systems
Is: industrial structure
SBM: slacks-based measurement
Urb: urbanization rate
